# Tracking Preeclampsia: The Role of Cerebral Biomarkers—A Narrative Review

**DOI:** 10.3390/ijms27020806

**Published:** 2026-01-13

**Authors:** Sakina Mustafa Vakhariya, Arshiya Shajahan, Rajani Dube, Subhranshu Sekhar Kar, Bellary Kuruba Manjunatha Goud, Swayam Siddha Kar

**Affiliations:** 1 Department of Obstetrics and Gynecology, RAK College of Medical Sciences, RAK Medical and Health Sciences University, Ras Al Khaimah P.O. Box 11172, United Arab Emirates; sakina.21901001@rakmhsu.ac.ae (S.M.V.); arshiya.21901039@rakmhsu.ac.ae (A.S.); 2 Department of Pediatrics, RAK College of Medical Sciences, RAK Medical and Health Sciences University, Ras Al Khaimah P.O. Box 11172, United Arab Emirates; subhranshu.kar@rakmhsu.ac.ae; 3 Department of Biochemistry, RAK College of Medical Sciences, RAK Medical and Health Sciences University, Ras Al Khaimah P.O. Box 11172, United Arab Emirates; manjunatha@rakmhsu.ac.ae; 4 Department of Obstetrics and Gynecology, School of Medicine, University of Lancashire, Preston Campus, Preston PR1 7BH, UK; swaekar35@gmail.com

**Keywords:** PE, neurological manifestations, cerebral biomarkers, neurofilament light chain, neuron-specific enolase, S100 Calcium Binding Protein B, tau protein

## Abstract

Preeclampsia (PE) is the onset of hypertension in pregnancy with systemic involvement; PE poses significant risks of cerebral complications, including eclampsia and long-term cognitive impairment. This review explores the potential of neurological biomarkers—neurofilament light chain (NfL), neuron-specific enolase (NSE), S100 Calcium Binding Protein B (S100B), and tau—as indicators of cerebral injury in PE. A literature search identified studies comparing biomarker levels in preeclamptic and healthy pregnancies. Findings reveal elevated plasma levels of NfL, NSE, S100B, and Tau in PE, with NfL showing the strongest association with blood–brain barrier dysfunction, cognitive symptoms, and disease severity. Variations between plasma and cerebrospinal fluid levels suggest impaired BBB integrity rather than increased central nervous system production. Despite promising correlations, limitations include small sample sizes, lack of standardized thresholds, and limited CSF data. While NfL emerges as a particularly promising marker for risk stratification, further research is needed to validate the clinical utility of these biomarkers in routine PE management.

## 1. Introduction

Preeclampsia (PE) is a multisystem hypertensive disorder of pregnancy characterized by new-onset hypertension (blood pressure ≥140/90 mmHg) after 20 weeks of gestation, accompanied by signs of maternal organ dysfunction (e.g., renal, hepatic, hematologic, or neurologic involvement) and/or fetal growth restriction. Proteinuria (≥300 mg/24 h) is a common but non-essential criterion [1]. PE affects approximately 2–8% of pregnancies worldwide, making it a leading cause of maternal and perinatal morbidity and mortality. The incidence varies by region, with higher rates in low- and middle-income countries due to limited access to prenatal care. In high-income countries, PE accounts for about 10–15% of maternal deaths, and it is a major reason for medically indicated preterm delivery. Risk factors for PE include first pregnancy, multiple gestation, obesity, advanced maternal age, pre-existing hypertension or diabetes, autoimmune diseases, and a history of PE in previous pregnancies [2].

Beyond its acute obstetric implications, PE is increasingly recognized as a disorder with significant neurological relevance. The pathophysiology involves abnormal placentation, systemic endothelial dysfunction, exaggerated inflammatory responses, and dysregulation of angiogenic factors, all of which contribute to widespread vascular injury [1,2]. Cerebral involvement is a particularly serious manifestation, driven by impaired cerebral autoregulation, endothelial activation, and increased blood–brain barrier (BBB) permeability. These changes predispose affected women to vasogenic edema, cerebral hypoperfusion, and hemorrhagic complications, which underpin conditions such as eclampsia and posterior reversible encephalopathy syndrome (PRES) [3]. One of the severe cerebral complications seen in PE is eclampsia, which is characterized by the onset of seizures in a woman with PE that cannot be attributed to other causes. Other complications, such as pulmonary edema, acute renal failure, and intracerebral hemorrhage, along with eclampsia, are major contributors to maternal mortality in women with PE. PE also increases the long-term risk of dementia, stroke, and seizures. However, predicting acute-phase complications remains challenging, as symptoms such as headaches and visual disturbances have poor prognostic accuracy [3]. Despite advances in obstetric care, neurological complications remain difficult to anticipate, and their onset can be sudden and catastrophic. The absence of objective biomarkers for cerebral complications in PE remains a critical gap in understanding and is increasingly drawing attention in current research [4].

Emerging evidence suggests that cerebral dysfunction in PE is not limited to the peripartum period. Women with a history of PE exhibit a higher lifetime risk of cognitive impairment, cerebrovascular disease, epilepsy, and dementia, suggesting that pregnancy may unmask or initiate long-term neurovascular vulnerability [3]. However, current clinical assessment relies largely on nonspecific symptoms and conventional imaging, which are neither sensitive nor practical for early risk stratification. This highlights an urgent need for reliable, minimally invasive biomarkers that reflect cerebral injury and BBB disruption in PE [4].

Neuroaxonal and glial biomarkers measured in peripheral blood have gained attention as potential tools to address this gap [4,5]. Neurofilament light chain (NfL), a structural protein of myelinated axons, is a well-established marker of neuronal injury and axonal degeneration in a wide range of neurological disorders [6]. Similarly, tau proteins reflect neuronal microtubule instability and neurodegeneration, while neuron-specific enolase (NSE) and S100 Calcium Binding Protein B (S100B) are associated with neuronal damage and astroglial activation, respectively [7,8,9,10]. Importantly, several studies have demonstrated elevated circulating concentrations of these biomarkers in women who develop PE, even before the onset of overt neurological symptoms [9,11]. NfL, tau, NSE, and S100B are established biomarkers of cerebral injury in neurological disorders. Studies in pregnant women suggest blood–brain barrier (BBB) alterations and neuroaxonal injury in PE, supported by higher plasma NfL (and its association with decreased trans-endothelial electrical resistance (TEER)) and increased permeability in an in vitro BBB model, than plasma from normal pregnancies or non-pregnant women [4,5].

Experimental and clinical data further support a mechanistic link between PE and cerebral injury. Plasma from women with PE has been shown to increase BBB permeability in in vitro models, accompanied by reduced TEER, indicating compromised BBB integrity [4,5]. Elevated levels of NfL, tau, NSE, and S100B in maternal circulation are consistent with neuroaxonal injury and astrocytic stress, likely secondary to endothelial dysfunction and BBB breakdown [4,5]. Collectively, these findings suggest that circulating cerebral biomarkers may provide objective insight into subclinical brain injury in PE and hold promise for improving risk stratification, monitoring, and understanding of neurological complications associated with this complex disorder.

Hence, this literature review aims to investigate changes in levels of cerebral biomarkers—NfL, tau, NSE, and S100B—in serum, plasma, Cerebrospinal fluid (CSF), and amniotic fluid among pregnant women at high risk of developing PE, those with early signs, established PE, and normal pregnancies. The review also examines the association between biomarker levels and BBB integrity, measured through trans-endothelial electrical resistance in an in vitro model. In addition, the analysis explores biomarker fluctuations across gestation and evaluates whether elevated CSF levels indicate cerebral injury and correlate with corresponding plasma concentrations.

## 2. Materials and Methods

A comprehensive literature review was conducted to explore the association between PE and neurological biomarkers, specifically neurofilament light chain (NfL), S100 Calcium Binding Protein B (S100B), neuron-specific enolase (NSE), and tau protein. A systematic search of PubMed and Web of Science was performed using the terms “Preeclampsia” AND (“NfL” OR “S100B” OR “NSE” OR “Tau”), restricted to original, English-language articles. Studies that examined serum or plasma or CSF or amniotic fluid levels of these biomarkers in women with PE compared to healthy pregnant controls were included. Key findings were extracted and synthesized, with results presented in tables and figures to summarize study characteristics, biomarker levels, and notable trends.

## 3. Results and Discussion

A total of 20 studies were included in the narrative review. Table 1, Table 2, Table 3 and Table 4 below summarize the studies included in our review, presenting statistical data on biomarker levels—NfL, NSE, S100B, and Tau—across three groups: preeclamptic women, healthy pregnant women, and non-pregnant women.

### 3.1. Cerebral Biomarkers—Several Biomarkers Have Been Proposed to Predict Neurological Involvement in Diseases

#### 3.1.1. Neurofilament Light Chain

Neurofilament light chain is a key structural protein of the neuronal cytoskeleton, predominantly expressed in large-caliber myelinated axons, where it contributes to axonal stability, caliber maintenance, and signal conduction. Axonal injury or degeneration leads to the release of NfL into the interstitial fluid, from where it diffuses into CSF and subsequently into peripheral circulation. Because of this direct relationship with axonal damage, NfL has emerged as a sensitive and relatively specific biomarker of neuroaxonal injury across a wide spectrum of neurological conditions [6]. Elevated NfL concentrations have been consistently demonstrated in neurodegenerative diseases, inflammatory disorders of the central nervous system, cerebrovascular disease, and acute brain injury, with levels reflecting both disease severity and progression [6].

The development of ultrasensitive analytical platforms, particularly single-molecule array (Simoa) technology (Quanterix Corporation, Lexington, MA, USA), has enabled reliable quantification of NfL in blood at concentrations previously detectable only in CSF. This advancement has transformed NfL into a clinically feasible, minimally invasive biomarker suitable for longitudinal monitoring and risk stratification [6]. In the context of pregnancy, this is especially relevant, as lumbar puncture is rarely justified, and blood-based biomarkers offer a safer alternative.

**Figure 1:**
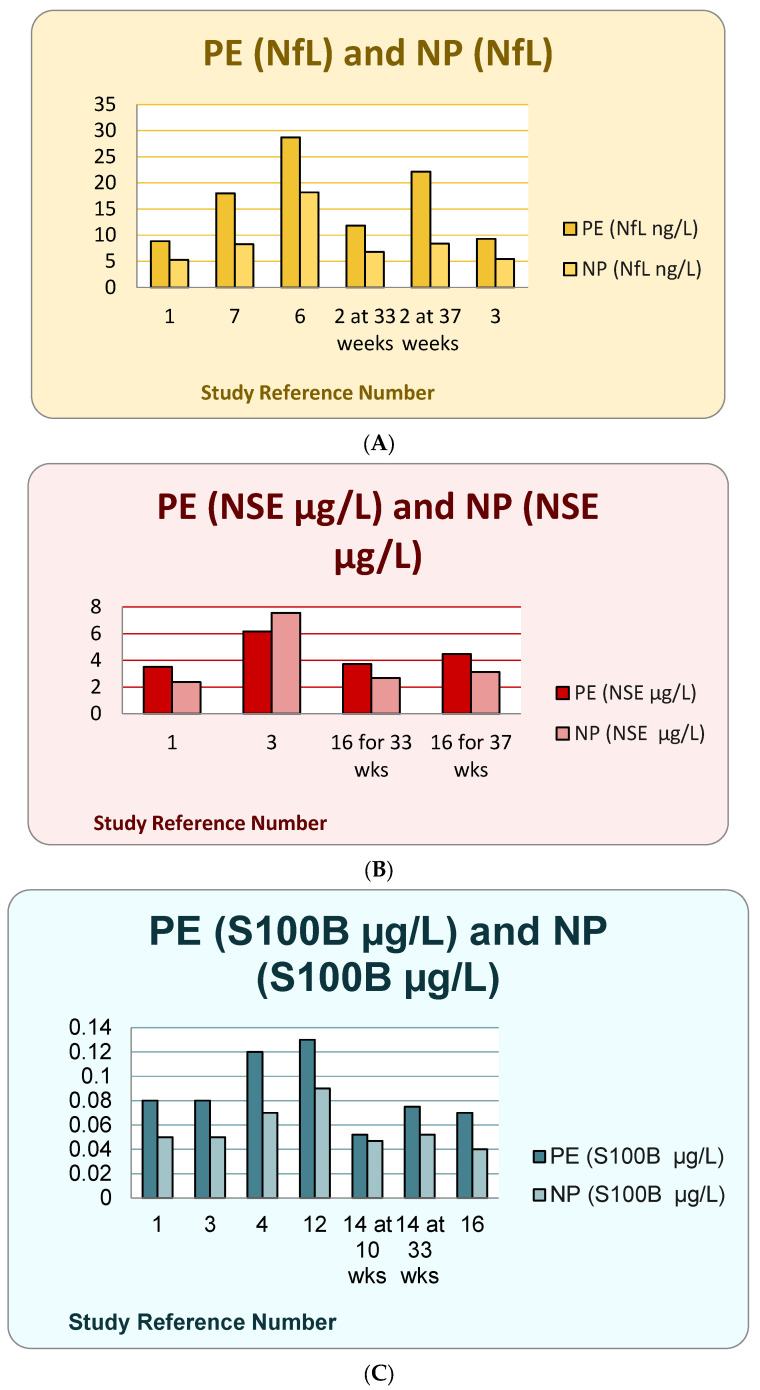

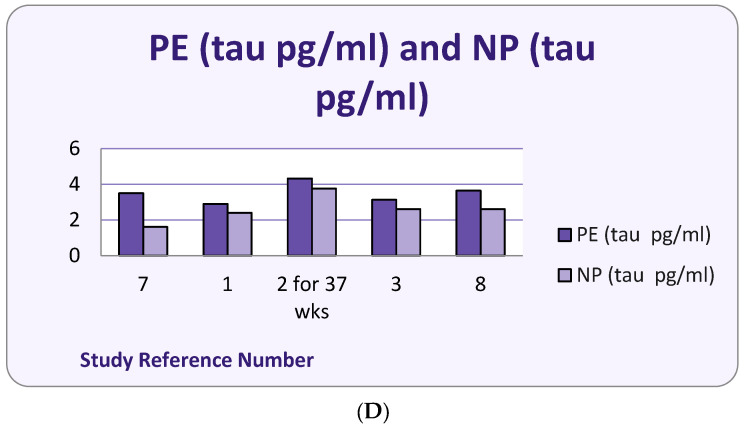
Showing changes of NfL (**A**), NSE (**B**), S100B (**C**), and Tau (**D**) of PE-PE and NP-normal pregnancy across the studies. (**A**): Across the studies, levels of NfL were higher in PE compared to NP. It was true for both gestational ages, 33 and 37 weeks. (**B**): The reported levels of NSE were higher in PE compared to NP in the majority of studies. Only one study reported higher levels in NP. (**C**): The levels of S100B were higher among PE compared to NP. However, the difference was less prominent in early pregnancy (10 weeks), compared to later gestation. (**D**): The levels of tau protein were higher among PE compared to NP across all studies.

In PE, accumulating evidence indicates that circulating NfL levels are significantly elevated compared with normotensive pregnancies, suggesting the presence of subclinical neuroaxonal injury [4,9] (Figure 1). Case–control and nested cohort studies have demonstrated increased plasma NfL concentrations both before and after clinical onset of PE, implying that neuronal injury may occur early in the disease process [9,10]. Experimental studies further support this association, showing that plasma from women with PE increases BBB permeability in vitro and correlates with higher NfL levels, reinforcing the link between endothelial dysfunction, BBB disruption, and axonal injury [4,5]. Importantly, elevated NfL has also been associated with severe neurological complications such as eclampsia and PRES, highlighting its potential prognostic value in identifying women at risk of cerebral involvement [12].

#### 3.1.2. Tau Protein

Tau is a microtubule-associated protein that plays a crucial role in maintaining neuronal cytoskeletal integrity, axonal transport, and synaptic function. Under physiological conditions, tau stabilizes microtubules within neurons; however, pathological processes such as oxidative stress, inflammation, and ischemia lead to abnormal phosphorylation, detachment from microtubules, and aggregation into insoluble forms [7]. These changes are central to the pathogenesis of Alzheimer’s disease and other tauopathies, making tau a well-established marker of neurodegeneration [7].

Elevated tau concentrations in CSF and blood reflect neuronal injury and ongoing neurodegenerative processes. Advances in ultrasensitive assays have enabled the detection of both total tau and phosphorylated tau isoforms in peripheral blood, facilitating their use in clinical and research settings [7]. In neurological disorders, circulating tau levels have been associated with cognitive impairment, disease severity, and long-term outcomes [7].

In PE, altered tau dynamics suggest that neuronal stress and degeneration may occur during pregnancy. Studies have demonstrated increased plasma tau concentrations in women who later develop PE, even in the absence of overt neurological symptoms [10]. Moreover, elevated tau levels have been reported in association with eclampsia and other neurological complications, supporting the concept of pregnancy-related cerebral injury [12]. Emerging evidence also links tau pathology in PE with later-life cognitive impairment, raising the possibility that PE may represent an early-life risk factor for neurodegenerative disease [13,14].

#### 3.1.3. Neuron-Specific Enolase

Neuron-specific enolase is a glycolytic enzyme predominantly expressed in neurons and neuroendocrine cells, where it plays a role in cellular energy metabolism. Under normal conditions, NSE is confined to the intracellular compartment, and gray matter exhibits the highest NSE content. The amount of NSE in the blood is at least 30 times lower than in the brain. However, neuronal injury, ischemia, hypoxia, or inflammation results in its release into extracellular fluid, CSF, and subsequent entry into the bloodstream, resulting from the breakdown of the BBB [8]. Because of this property, NSE has long been used as a biomarker of neuronal cell damage in conditions such as traumatic brain injury, stroke, hypoxic–ischemic encephalopathy, and cardiac arrest [8].

Clinically, NSE levels correlate with the extent of neuronal injury and have prognostic value, particularly in critically ill patients [15]. Persistently elevated NSE concentrations are associated with poor neurological outcomes and increased mortality [8]. Importantly, NSE reflects neuronal cell body damage rather than axonal injury, making it complementary to biomarkers such as NfL.

In PE, several studies have demonstrated elevated plasma NSE levels during pregnancy in women who develop the disorder compared with normotensive controls [10,16]. These findings suggest that neuronal injury occurs even in the absence of clinically apparent neurological complications. Elevated NSE concentrations have also been associated with severe forms of PE and neurological manifestations such as seizures, supporting the role of NSE as a marker of cerebral involvement [12]. The presence of increased NSE may reflect hypoxic–ischemic injury secondary to cerebral vasoconstriction, endothelial dysfunction, and impaired autoregulation characteristic of PE [3]. The consistent elevation of NSE across different cohorts highlights its potential utility as a readily measurable biomarker of neuronal injury in PE. When interpreted alongside NfL and tau, NSE may contribute to a multidimensional assessment of cerebral damage, capturing distinct but overlapping aspects of neuronal pathology [10,12].

#### 3.1.4. S100B

S100B is a calcium-binding protein predominantly expressed and secreted by astrocytes within the central nervous system. At physiological concentrations, S100B plays a role in neurotrophic signaling, synaptic plasticity, and astrocyte–neuron communication. However, elevated extracellular levels are indicative of astrocytic activation, glial injury, or BBB disruption [9]. Because S100B can cross a compromised BBB, increased serum concentrations are widely interpreted as a marker of cerebral injury and BBB permeability.

S100B is one of the most extensively studied cerebral biomarkers in pregnancy-related disorders. Numerous studies have reported significantly elevated serum and plasma S100B levels in women with PE, particularly in severe and early-onset disease [11,17,18,19]. Increased S100B concentrations have also been detected before the clinical onset of PE, suggesting early astrocytic stress and BBB dysfunction [19]. These findings are consistent with experimental data demonstrating increased BBB permeability in PE and its association with endothelial dysfunction [4,5].

Importantly, elevated S100B levels have been linked to neurological symptoms, cognitive impairment, and radiological evidence of cerebral involvement in women with PE and eclampsia [12,14]. Studies have also shown increased S100B expression in placental tissues and amniotic fluid in PE, indicating a systemic inflammatory and neurovascular response rather than isolated cerebral pathology [11].

Given its sensitivity to BBB disruption and astroglial injury, S100B represents a valuable biomarker for identifying cerebral involvement in PE. When combined with neuronal and axonal markers such as NSE and NfL, S100B enhances the ability to characterize the spectrum of brain injury associated with PE and may help identify women at the highest risk of neurological complications [9,10,12].

### 3.2. Biomarker Trends in Healthy vs. Preeclamptic Pregnancies

Longitudinal studies on healthy pregnancies show that NfL levels rise at week 37 [10], S100B remains unchanged [11], and tau levels decrease over time [10]. In contrast, women who develop PE exhibit significantly higher levels of NfL, S100B, and tau [10,11] at weeks 28, 33, and 37 compared to healthy controls. In addition, NSE levels remain consistently elevated throughout pregnancy in women with PE, whereas they decline over time in healthy pregnancies [6]. These trends suggest that altered trajectories of these biomarkers may reflect early neuroinflammatory or neuroaxonal changes associated with PE development.

### 3.3. Plasma and CSF Biomarker Differences

Higher concentrations of NfL are observed in both plasma [3,4,5,9,10,12] and CSF [3] of women with PE when compared to normal pregnant and non-pregnant women. Similarly, S100B levels are elevated in the plasma [3,4,16,17,18] and amniotic fluid [13,14] of preeclamptic women, while CSF concentrations of S100B show no significant difference between groups [3]. For NSE and tau, plasma levels are higher in PE [1,3,4,12], but both biomarkers are found in lower concentrations in the CSF of affected women [3]. These findings point toward possible BBB dysfunction, where increased plasma levels may reflect leakage or altered transport mechanisms rather than increased CNS production.

### 3.4. NfL as a Potential Risk Stratification Biomarker of PE

NfL levels rise with maternal age, with a more marked increase in women with PE, indicating that NfL could serve as a predictive marker for the condition, particularly in older mothers, with performance comparable to angiogenic factors [9]. Evidence shows that plasma NfL levels also correlate with impaired cognition in cases of eclampsia and PE complicated by pulmonary edema, suggesting that neuroaxonal injury may contribute to cognitive decline in these conditions (Table 1). 

**Table 1:** Evidence on NfL.

Study Type, Year and Reference	Study Group	Results	Summary
Case-control study—2022 [4]	PE = 28, NP = 28, Non-P = 16	NfL higher in PE than in NP (8.85 vs. 5.25 ng/L, *p* < 0.001); NfL higher in PE compared with non-pregnant women (8.85 vs. 5.65 ng/L, *p* < 0.001) NfL higher in PE with severe headache (11.65 vs. 7.40 ng/L, *p* = 0.024).	The levels of NfL in plasma were significantly higher in PE than in NP. Significantly higher plasma concentrations of NfL in PE with severe headache than with mild or no headache. No associations between visual disturbances and NfL levels.
Case-control study—2022 [12]	Serum: NP = 28 and PE = 146, including subgroups with and without complications. CSF: PE = 8 and NP = 7	2.18-fold higher plasma NfL (95% CI, 1.64–2.88) in PE vs. NP. HELLP group 1.64-fold higher plasma NfL (95% CI, 1.06–2.55) No difference in NfL with/without pulmonary edema	Women with PE had significantly higher levels of NfL in plasma than those with NP. This was also seen in women with HELLP, but not with PE complicated with pulmonary edema.
Prospective, longitudinal study—2018 [9]	Total = 197, PE = 60	NfL higher in pregnancy in those who developed PE vs. who did not (28.7 pg/mL vs. 18.2 pg/mL, <0.001) The discriminatory accuracy of NfL in the ROC curves analysis of the overall group was 0.68, and in those over 36 years of age is 0.7.	NfL predicts PE particularly in older women (>36 years). It may serve as an early indicator of PE-induced neuronal changes. Maternal age amplified NfL levels in PE.
Case-control study (longitudinal)—2018 [11]	PE = 16, NP = 36	NfL higher in PE at week 33 (11.85 vs. 6.80; *p* < 0.05) and week 37 (22.15 vs. 8.40; *p* < 0.01). At 33 weeks AUC for NfL 0.62 (0.17–1.00)	NfL increased at the end of pregnancy in women developing PE in contrast to NP. It may show cerebral involvement before onset of disease.
Case-control study—2021 [3]	severe PE = 15, NP = 15	PE had ↑ serum NfL (9.29 vs. 5.44 pg/mL, *p* < 0.001) and CSF NfL (396 vs. 336 pg/mL, *p* < 0.01) compared to NP.	Increased serum and CSF levels of NfL show neuroaxonal injury in PE, even in the absence of clinically evident neurological complications.
Cross-sectional study—2023 [19]	Eclampsia = 49, PE with pulmonary edema = 16, uncomplicated PE = 22, NP = 18	NfL correlated with cognitive impairment in eclampsia and PE + pulmonary edema (r = −0.37, *p* = 0.009 and r = −0.56, *p* = 0.025).	No correlation between impaired cognitive function and NfL in PE without pulmonary edema, HELLP or NP. Acute neuroaxonal injury associated with cognitive impairment in PE.
Retrospective observational cohort study—2021 [20]	Obstetric PRES = 123 and non-PRES = 99	NfL levels were significantly ↑ in the PRES group than in the non-PRES group (*p* < 0.0001). The discriminatory accuracy of NfL in ROC curve analysis was 0.7664.	NfL level was significantly correlated with edema severity (*p* < 0.0001), and a poorer pregnancy outcome

Note: NP = Normal Pregnancy; Non-P = Non-pregnant; PE = Preeclampsia; PRES = Posterior Reversible Encephalopathy Syndrome; HELLP = Hemolysis, Elevated Liver Enzymes, Low Platelets; ROC = Receiver Operating Characteristic; AUC = Area under curve.

However, no such correlation is observed in women with uncomplicated PE or normotensive pregnancies, and tau does not show any correlation with cognitive impairment in any group [19].

Collectively, the available evidence supports NfL as a sensitive marker of neuroaxonal stress in PE, with particular relevance to disease severity rather than uncomplicated hypertension alone. Across study designs—prospective, longitudinal, case–control, and retrospective—NfL levels are consistently higher in PE than in normotensive or non-pregnant controls, with effect sizes that remain significant after accounting for gestational age and maternal characteristics [3,4,9,10,12]. Importantly, studies measuring both CSF and plasma demonstrate parallel elevations, strengthening the biological plausibility that circulating NfL reflects central neuroaxonal injury rather than peripheral confounding [3,12]. The stronger association of NfL with neurological complications—such as eclampsia, PRES, and cognitive impairment—compared with uncomplicated PE suggests a threshold phenomenon, whereby rising NfL identifies women in whom endothelial dysfunction and BBB breakdown have progressed to overt cerebral injury [19,20]. The absence of correlation between NfL and pulmonary edema alone further supports its specificity for neurological involvement rather than systemic disease severity [12]. However, age-related increases and inter-individual variability underscore the need for gestation- and age-adjusted reference ranges before clinical implementation [9].

### 3.5. PE and NSE

The evidence on NSE in PE suggests modest but consistent neuronal involvement, with findings varying by biological compartment and disease severity (Table 2). 

**Table 2:** Evidence on NSE.

Study Type, Year and Reference	Study Group	Results (NSE)	Summary
Case–control study—2022 [4]	PE = 28, NP = 28, Non-P = 16	NSE in PE is higher compared to NP: 3.50 µg/L vs. 2.37 µg/L (*p* < 0.001)	NSE levels were significantly higher in PE compared to NP. However, NSE did not relate to severity of headache, or visual disturbances.
Case–control study—2021 [3]	Severe PE = 15, NP = 15	CSF NSE was lower in PE than in NP (6.16 vs. 7.56 µg/L, *p* < 0.05), with no difference in plasma levels between them.	NSE in CSF was higher in PE complicated by cerebral edema, which may be due to larger cerebral insult in cases with edema.
Nested case–control study—2016 [16]	Total = 469, PE = 16 and NP = 36	At week 33, NSE is higher in PE than NP (3.72 µg/L vs. 2.67 µg/L, *p* = 0.05). At week 37, NSE was higher in PE; it declined in NP (4.47 µg/L vs. 3.12 µg/L; *p* < 0.001).	In women developing PE, NSE levels were high throughout pregnancy. In NP, it decreases with progress of pregnancy. It suggests failure to decline in PE.

Note: NP = Normal Pregnancy; Non-P = Non-pregnant; Preeclampsia = PE.

Plasma NSE levels are significantly elevated in women with PE compared with normotensive and non-pregnant controls, particularly in late gestation, indicating progressive neuronal stress as the disease advances [4,16]. Longitudinal data demonstrate a gestational rise in NSE in PE, contrasting with declining levels in normotensive pregnancies, supporting its association with pathological rather than physiological pregnancy-related changes [16]. In contrast, reduced cerebrospinal fluid NSE in severe PE, despite unchanged plasma levels, may reflect altered CNS release dynamics or compartmental redistribution in advanced disease [3].

### 3.6. PE and S100B

The collective evidence consistently supports S100B as a sensitive biomarker of cerebral involvement in PE, reflecting astrocytic activation and BBB dysfunction rather than overt neuronal loss. Multiple case–control studies demonstrate significantly elevated serum or plasma S100B levels in women with PE compared with normotensive pregnancies, with reproducible effect sizes across different populations [3,4,17,21]. Importantly, longitudinal data show that S100B levels rise progressively during pregnancy in women who develop PE, while remaining stable in normotensive controls, indicating that S100B elevation is disease-related rather than gestation-dependent [14].

Disease severity appears to be a key determinant of S100B elevation. Higher concentrations are consistently observed in severe compared with mild PE and are strongly associated with neurological symptoms, including visual disturbances and central nervous system involvement [13,17,22]. The identification of clinically relevant thresholds, with elevated S100B conferring a markedly increased risk of CNS symptoms and HELLP syndrome, underscores its potential prognostic utility [17]. The absence of significant differences in CSF S100B despite elevated serum levels suggests that peripheral S100B primarily reflects BBB leakage rather than direct intrathecal production [3]. Additionally, increased S100B in amniotic fluid highlights a broader fetoplacental and systemic inflammatory response in PE [11]. Collectively, these findings position S100B as a robust marker of neurovascular dysfunction in PE with potential value for early risk stratification and severity assessment (Table 3). 

**Table 3:** Evidence on S100B.

Study Type, Year and Reference	Study Group	Results	Summary
Case–control study—2022 [4]	PE = 28, NP = 28, non-P = 16	Plasma S100B higher in PE compared to NP (0.08 µg/L vs. 0.05 µg/L, *p* < 0.01)	S100B levels were significantly higher in PE compared to NP. It did not relate to severity of headache, or visual disturbances.
Case–control study—2021 [3]	PE = 15, NP = 15	Serum S100B higher in PE than NP (0.08 vs. 0.05 µg/L, *p* < 0.01), but no difference was found in the CSF concentration between the two.	It can be either due to extracerebral levels contributing predominantly to the serum, or S100B produced in astrocytic end-feet close to the BBB is secreted in higher amounts into the bloodstream due to BBB injury and depleted from the CNS.
A cross-sectional case–control study—2014 [22]	PE = 53, NP = 58	Plasma S100B higher in PE compared NP (0.12 µg/L vs. 0.07 µg/L, *p* < 0.001) and significantly linked to visual disturbances (*p* < 0.05).	Higher levels of S100B in PE and association with visual disturbances reflects possible CNS affection in women with PE.
Prospective case–control study—2015 [17]	Severe PE = 27, NP = 36	S100-B levels were higher in severe PE (0.09 vs. 0.13 µg/L, *p* = 0.025). For predicting PE, at levels ≥ 0.0975 µg/L, sensitivity and specificity were found to be 81.4% and 58.3%. At levels ≥ 0.0975 µg/L, 12.75-fold ↑ risk of CNS symptoms and a 3.27-fold ↑ risk of HELLP syndrome. AUC value for S100-B was calculated as 0.712.	S100B levels may be a potential marker in severe PE for the severity of hypoperfusion both in the placenta & brain, leading to subsequent risk of organ failure.
Case control study—2024 [21]	PE = 9, NP = 13	S100B levels were ↑ in PE than in normal pregnancies (*p* < 0.05). ROC curve analysis showed that S100B detected by SPRi method had a cut-off level of 181 ng/mL with a sensitivity of 100%, a specificity of 84.6%, and a Youden index of 0.846	S100B levels detected by SPRi in maternal blood can indicate early-onset severe preeclampsia and perinatal brain injury
Case–control within a longitudinal study cohort—2012 [18]	Total = 469, PE = 16, NP = 37	Plasma S100B levels remained unchanged in NP but increased in PE from weeks 10 to 37 (0.052 vs. 0.075; *p* < 0.05). Women with PE had higher levels of S100B than NP at weeks 33 and 37 (*p* = 0.047 and *p* = 0.010, respectively).	Increased S100B level is seen in women developing PE compared to NP weeks before symptoms of PE. Increased S100B in PE might be secondary to cerebral vascular damage. S100B is a potential peripheral biomarker reflecting cerebral involvement in PE.
Case–control study—2012 [13]	NP = 15, Mild PE = 12, Severe PE = 34	Severe PE group demonstrated higher S100B levels (0.20 ± 0.19), as compared with mild PE (0.07 ± 0.05) or NP (0.04 ± 0.05)	Elevated serum S100B levels in severe PE suggest neural damage, and subsequent astrocytic release of S100B is independent of progression to eclampsia.
Case–control study—2006 [11]	PE = 7, NP = 35	Amniotic fluid S100B levels were higher in PE than NP. Concentration of S100B in amniotic fluid in PE (0.47 μg/L) & normotensive IUGR (0.60 μg/L) were significantly higher than in NP (0.18 μg/L). *p* < 0.05. At 33 weeks, AUC for S100B 0.95 (0.82–1.00)	CNS is affected in PE early, even in mild to moderate disease. AUC for combined marker was >0.7 at GA week 25, reflecting early CNS involvement months before the onset of clinical disease

Note: NP = Normal Pregnancy; Non-P = Non-pregnant; PE = Preeclampsia; HELLP = Hemolysis, Elevated Liver enzymes, Low Platelets; IUGR = Intrauterine Growth Restriction; BBB = blood–brain barrier; CNS = Central nervous system; GA = Gestational age.

### 3.7. PE and Tau Protein

The evidence indicates that tau is elevated in PE, particularly in association with disease severity and neurological complications, although its behavior differs across biological compartments (Table 4).

**Table 4:** Evidence on Tau protein.

Study Type, Year and Reference	Study Group	Results	Summary
Case–control study 2022 [12]	PE = 146, NP = 28	PE had 2.17-fold higher levels of tau vs. controls, (95% CI, 1.49–3.16). PE with neurological complications levels of tau (2.99-fold higher; 95% CI, 1.92–4.65). HELLP group tau levels (4.44-fold higher; 95% CI, 1.85–10.66)	Neurologic complications (eclampsia, cortical blindness, and stroke) in PE linked to higher levels of tau protein. tau protein was also increased in other neurologic complications compared with women with eclampsia only.
Case–control study 2022 [4]	PE = 28, NP = 28, Non-P = 16	Tau higher in PE vs. NP (2.90 vs. 2.40 ng/L) *p* < 0.05	Tau levels were significantly higher in PE compared to NP. However, NSE did not relate to severity of headaches, or visual disturbances.
Cross-sectional 2023 [14]	PE = 68, NP = 30, Non-P = 48	Higher P-tau181 in PE vs. both control groups (both *p* < 0.05)	According to the ROC curve, P-tau 181 had statistical significance in predicting the ability of cognizance (*p* < 0.05). High serum P-tau181 can be used as an indicator for non-invasive assessment of cognitive functional impairment in PE patients.
Nested case–control study within a longitudinal study cohort 2018 [11]	PE = 16, NP = 36	In NP, there was a reduction in tau in gestational week 25, 28, 33 (*p* < 0.05), 37 (*p* = 0.06) compared to week 10. In PE, levels of tau were similar to NP at all GA except in week 37, where PE had higher tau than NP (*p* < 0.05). At 33 weeks AUC for tau 0.57 (0.19–0.95).	CNS is affected in PE early even in mild to moderate disease. AUC for combined marker was >0.7 at GA week 25, reflecting early CNS involvement months before the onset of clinical disease.
Case–control study 2021 [3]	severe PE = 15, NP = 15	CSF tau was lower in PE vs. controls (228 vs. 315 pg/mL, *p* < 0.05); plasma tau levels were higher in PE, but not significant (3.13 pg/mL vs. 2.32 pg/mL)	Extracellular levels of tau are regulated by neuronal-activity-dependent release of the protein, determining CSF concentrations. Reduced CSF concentrations of tau in PE could reflect reduced neuronal activity.
Cross-sectional study 2023 [19]	Eclampsia = 49, PE = 38, NP = 18	PE shows higher plasma concentrations than NP, (3.64 pg/mL vs. 2.61 pg/mL, <0.001).	No correlation between impaired cognitive function and tau in PE without pulmonary edema, HELLP or NP.

Note: NP = Normal Pregnancy; Non-P = Non-pregnant; CSF = Cerebrospinal fluid; AUC = Area under curve; CI = Confidence interval; P tau = Phosphorylated tau.

Multiple studies report significantly higher plasma tau concentrations in women with PE compared with normotensive and non-pregnant controls, supporting the presence of neuronal stress or injury in PE [4,12,14,19]. Longitudinal data suggest that tau elevation occurs later in gestation, becoming apparent near term rather than early in pregnancy, which may limit its value as an early predictive marker [11]. Importantly, higher tau levels are linked to neurological complications, reinforcing its association with cerebral involvement rather than uncomplicated disease [12]. In contrast, cerebrospinal fluid tau concentrations are paradoxically lower in severe PE despite elevated circulating levels, possibly reflecting altered intrathecal dynamics, impaired neuronal release, or increased peripheral clearance in advanced disease states [3]. Notably, unlike NfL, tau does not correlate with cognitive impairment in PE or eclampsia, suggesting that tau reflects acute neuronal stress rather than functional neurological outcomes [19].

### 3.8. Endothelial and Angiogenic Pathways, Blood–Brain Barrier Disruption, & Biomarker Elevation

Preeclampsia is characterized by systemic endothelial dysfunction with well-established angiogenic and molecular mechanisms that extend to the cerebral circulation [23,24]. In the CNS angiogenesis, the homeostatic control of the endothelial phenotype resides in cells in close proximity to the vessels, such as astrocytes, pericytes, neurons, and microglia. Such interactions shape the unique characteristics of the BBB [25]. Vascular endothelial growth factor (VEGF) is a vasodilator, and VEGF receptor 2 (VEGFR2) is essential for maintaining BBB integrity through regulation of endothelial tight junctions and vascular permeability [25]. Pregnancy significantly affects cerebral hemodynamics, decreasing vascular resistance, increasing cerebral blood flow, and BBB permeability [26]. Excessive release of soluble fms-like tyrosine kinase-1 (sFlt-1) from human placenta also antagonizes VEGF signaling, resulting in impaired VEGF–VEGFR2 activation [23,26]. Experimental and clinical evidence demonstrates that altered VEGFR2 signaling in PE is associated with increased BBB permeability and cerebral edema, providing a direct mechanistic link between angiogenic imbalance and cerebral vascular dysfunction [26].

Angiopoietin dysregulation further destabilizes the cerebral endothelium. A shift toward angiopoietin-2 dominance promotes endothelial junctional loosening, vascular inflammation, and vasogenic edema—pathophysiological features frequently observed in severe PE and PRES [27,28,29]. In parallel, nitric oxide dysregulation driven by oxidative stress and endothelial nitric oxide synthase uncoupling impairs cerebrovascular autoregulation, thereby increasing susceptibility to hyperperfusion injury and BBB breakdown [30].

These endothelial and angiogenic disturbances provide a mechanistic basis for the elevation of circulating cerebral biomarkers. Clinical and experimental studies demonstrate increased BBB permeability in PE, accompanied by elevated plasma and CSF concentrations of NfL, tau, S100B, and NSE, particularly in women with cerebral edema or neurological manifestations [9]. NSE is highly concentrated in cortical gray matter, and its release into CSF and blood reflects neuronal membrane disruption and BBB failure following cerebral injury [31]. Similarly, S100B, produced predominantly in astrocytic end-feet adjacent to the BBB, may preferentially enter the systemic circulation when barrier integrity is compromised rather than accumulate in CSF [31,32].

Integrating angiogenic imbalance, endothelial dysfunction, and BBB disruption strengthens the mechanistic framework linking PE to cerebral biomarker level changes and increased neurological risk, including eclampsia and PRES [28].

### 3.9. Cognitive and Neurological Manifestations

Elevated levels of S100B in PE are significantly associated with visual disturbances [20]. Notably, S100B concentrations ≥0.0975 µg/L are linked to a 12.75-fold increased risk of central nervous system symptoms and a 3.27-fold increased risk of HELLP syndrome, highlighting its potential role as a marker of severe disease [18]. NfL concentrations are also higher in preeclamptic women experiencing severe headaches compared to those with mild or no symptoms [4]. Furthermore, among patients with PE or eclampsia, NfL levels are significantly elevated in those diagnosed with obstetric PRES, supporting its role as both a diagnostic and prognostic biomarker related to neurological involvement and adverse pregnancy outcomes [20]. A recent study also highlights the novel role of the Pin1-cis P-tau-ApoE axis in the development of PE, and propagation of cis P-tau-mediated abnormal protein aggregation (tauopathy) from the placenta to cerebral tissues later in life, leading to neurodegenerative conditions. In the case of PE, proteinopathy/tauopathy may interrupt trophoblast differentiation and induce cell death, similar to the events occurring in neurons [33]. Mechanistic studies further suggest placental and epigenetic involvement in tau dysregulation. Altered methylation of tau-related genes in placental tissue and abnormal tau signaling pathways have been described in PE and long-term neurological sequelae [34,35]. Together, these findings position tau as a biomarker reflecting both acute neuronal injury and potential long-term neurodegenerative risk in women affected by PE. Collectively, these findings indicate that NfL, tau, NSE, and S100B may serve as useful biomarkers for detecting and monitoring cerebral involvement in PE [3].

This is a comprehensive review that includes the narratives of multiple biomarkers for neurological involvement in PE. While emerging biomarkers such as NfL, tau, NSE, and S100B show promise in detecting neurological involvement in PE, several limitations remain. First, there is a lack of large-scale longitudinal studies and follow-up studies linking the levels of these biomarkers across different stages of pregnancy and postpartum to persistent neurological or cognitive outcomes to better understand their predictive value. Establishing precise threshold levels for at-risk populations is also necessary to improve diagnostic accuracy. Current research is limited regarding the evaluation of these biomarkers in alternative biological fluids such as amniotic fluid and CSF, which could offer additional diagnostic insights. Moreover, comparative studies are needed to evaluate the diagnostic performance of these neuro-injury markers against established PE detection methods, including blood pressure monitoring, proteinuria, symptomatology, homocysteine levels, and the sFlt-1/PlGF ratio. Clinically, lumbar puncture is not routinely performed in patients with suspected PE, limiting access to CSF-based data. Additionally, in vitro BBB models used in preclinical studies do not fully replicate the complexity of the human BBB, which may affect translational validity. Elevated plasma NfL levels can also be influenced by multiple factors, including generalized neuroaxonal injury or BBB disruption due to circulating anti-angiogenic or inflammatory molecules, making interpretation more complex. Lastly, there is considerable heterogeneity of the type of studies and PE phenotypes across the studies, including variation in disease onset, severity, and presence of neurological complications, which may contribute to variability in biomarker levels and limit generalizability and causal inference across all PE subtypes. Future research should aim to address these gaps to optimize the clinical utility of these biomarkers in PE.

## 4. Conclusions

NfL, S100B, NSE, and tau are biomarkers associated with neurological manifestations in PE. However, the assumption that plasma biomarkers are associated with BBB dysfunction, rather than increased CNS production, remains to be explicitly proven. Across diverse study designs, NfL levels in plasma/serum (and CSF) are consistently elevated, particularly in severe phenotypes, HELLP, eclampsia, and PRES, supporting it as a marker of neuroaxonal injury in PE. NfL shows potential clinical utility for early detection of neurological complications and risk stratification, with stronger discriminatory value in older women (>36 years). The current evidence for other markers is still uncertain. Their greatest value may lie in research settings or in risk stratification, especially for women with a history of PE or those presenting with additional risk factors. Further large-scale, longitudinal studies are needed to validate their clinical relevance and to establish standardized thresholds that could guide their integration into routine obstetric care.

## Data Availability

No new data is generated by this review. All the information is part of this manuscript.
